# Oral verruciform xanthoma (OVX) of lower labial mucosa, presenting as a plaque in tobacco users: an unusual case report with literature review

**DOI:** 10.3332/ecancer.2024.1796

**Published:** 2024-11-13

**Authors:** Sandhya Tamgadge, Treville Pereira, Aditi Vaidya, Vishal Punjabi

**Affiliations:** 1 Department of Oral & Maxillofacial Pathology and Microbiology, D.Y. Patil University School of Dentistry, Sector 7, Nerul, Navi Mumbai 400706, Maharashtra, India; 2 Department of Oral Pathology and Microbiology, D.Y. Patil University School of Dentistry, Sector 7, Nerul, Navi Mumbai 400706, Maharashtra, India

**Keywords:** oral verruciform xanthoma, lower lip, white plaque, tobacco, CD 68

## Abstract

Oral verruciform xanthoma (OVX) is an uncommon lesion known for its wart-like appearance, primarily affecting the oral mucosa. This case report delves into a distinctive presentation of OVX, a rare benign lesion typically characterised by its manifestation as a white plaque in the oral cavity. Clinical features, histological findings, pathogenesis and their implications in the context of differential diagnosis has been discussed. The patient had a history of regular tobacco use, and despite initially presenting as a white plaque, the histopathological and immunohistochemical features strongly suggested verruciform xanthoma.

## Introduction

Oral verruciform xanthoma (OVX) is an infrequent, benign lesion of the oral mucosa, first described by Shafer [1] in 1971. Typically, OVX presents as a well-demarcated, elevated and verrucous lesion [2]. However, this case report highlights an atypical presentation as smooth white plaques resembling leukoplakia, creating a diagnostic dilemma.

OVX has been reported in various oral locations, including the gingiva, hard palate, tongue and buccal mucosa [3, 4]. Notably, its occurrence on the lower labial mucosa is extremely rare. Belknap et al [3] reported that only 1.86% of verruciform xanthoma cases occur on the lower lip.

Several retrospective studies have contributed to our understanding of OVX. Santiago et al [5] analysed 90 cases, while Belknap et al [3] presented a substantial series of 212 cases. Yu et al [4] conducted a clinicopathological study of 15 cases, and Tamiolakis et al [2] reported 13 new cases. Barrett et al [6] presented (Table 1) eight typical and three anomalous cases, underscoring the diverse presentations of OVX.

The pathogenesis of OVX remains unclear, with various associated factors proposed. These include chronic trauma [7], inflammatory responses [2, 8] abnormal lipid metabolism [9] and genetic predispositions [10]. While some studies have explored a possible link with HPV, Hu et al [11] found no evidence of HPV in their report of three cases. The pathophysiology of OVX may involve MCP1/CCR2-mediated recruitment of foamy macrophages and lysosomal engulfment of epithelial lipids under T lymphocyte regulation [12]. Necrosis of foamy macrophages and macrophage-dependent debris may perpetuate the lesion [12].

Our case is unique due to its atypical location on the labial mucosa and its presentation resembling leukoplakia. This unusual occurrence highlights the importance of histopathological examination for a definitive diagnosis, as clinical appearance alone can be misleading in such cases.

## Case presentation

**Figure 1.**
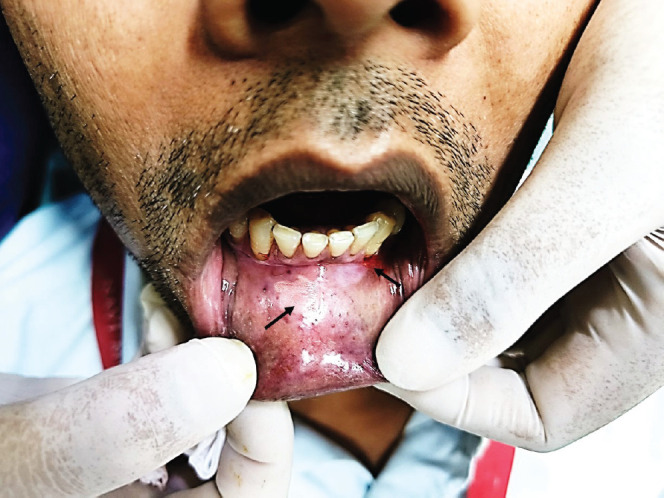
Clinical photograph showing two lesions on lower labial mucosa mimicking leukoplakia.

**Figure 2.**
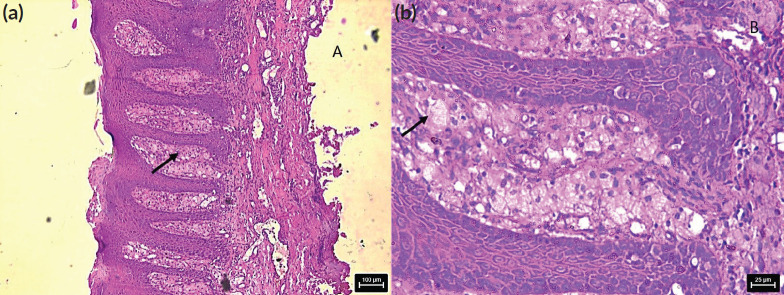
Photomicrograph shows H&E stained section with foamy cells in stroma, (a): low magnification and (b): high magnification.

**Figure 3.**
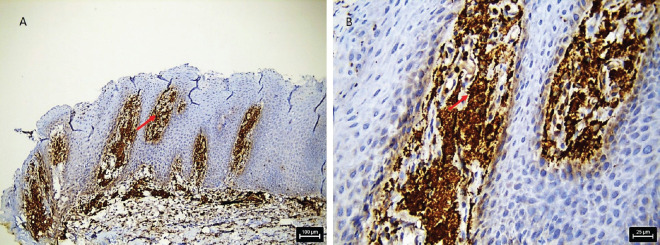
Photomicrograph shows IHC stained section with foamy cells in stroma positive with CD 68, (a): low magnification and (b): high magnification.

**Figure 4.**
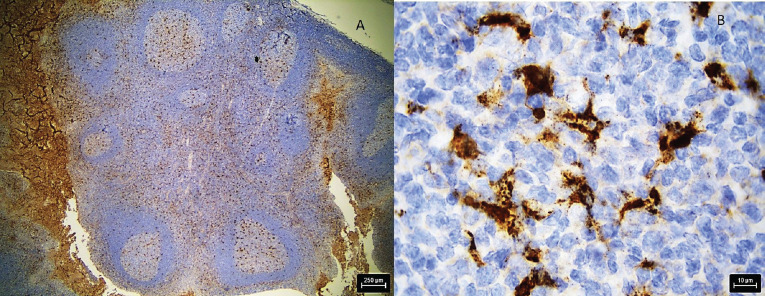
Photomicrograph shows IHC stained section of positive control shows positive staining with CD 68, (a): low magnification and (b): high magnification.

**Figure 5.**
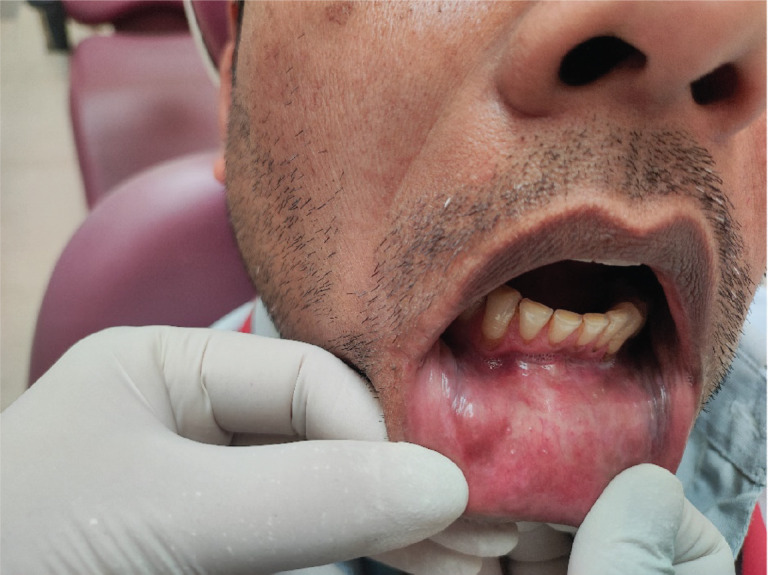
Postoperative follow-up after 6 months.

A 36-year-old Indian male from a low socioeconomic background presented with two asymptomatic white plaques on the lower labial mucosa. The larger plaque measured 1.5 × 1.5 cm, while the smaller one was 0.6 × 0.5 cm (Figure 1). Both were non-scrapable with slightly non-warty, smooth surfaces and no palpable induration or pain. The patient reported a 3-year history of tobacco use but had no significant medical history, allergies or previous oral lesions. Clinical examination posed a diagnostic challenge in differentiating these plaques from leukoplakia or lichen planus. An excisional biopsy was performed, revealing verruciform xanthoma cells with lipid-laden foamy macrophages (Figure 2). Immunohistochemical staining with CD68 confirmed the diagnosis of OVX (Figures 3 and 4). A lipid profile showed slightly low very low-density lipoprotein (VLDL) at 30 mg/dl. Follow-up at 1 week and 6 months post-biopsy demonstrated satisfactory healing (Figure 5). This case highlights an atypical presentation of OVX as white plaques, emphasising the importance of histopathological examination in diagnosing oral lesions.

## Discussion

Our case of OVX presents several noteworthy features when compared to the existing literature. While OVX typically manifests as a papillary or verrucoid mass, with the gingiva being the most common site [7], our patient exhibited two smooth-surfaced and Belknap *et al* [3] white plaques on the lower labial mucosa. This presentation aligns with the findings of Toida and Koizumi [13] and Cebeci *et al* [14] who reported cases of OVX on the lip, highlighting the clinical diversity of this condition.

The location of our case on the labial mucosa is particularly rare, contrasting with the more common occurrence of masticatory mucosa reported in 70% of cases according to Tamiolakis *et al* [2]. This atypical presentation posed a diagnostic challenge, initially resembling leukoplakia, which emphasizes the importance of considering OVX in the differential diagnosis of oral white lesions.

Histologically, our case aligned with the characteristic features of OVX, showing aggregates of foamy histiocytes within the connective tissue papillae as mentioned by Barrett *et al* [6]. The positive CD68 immunohistochemical staining further confirmed the diagnosis, consistent with previous studies Iamaroon and Vickers [9].

Our patient’s history of tobacco use is noteworthy. While OVX is not directly linked to tobacco use, this factor warrants careful consideration, especially given the lesion’s resemblance to leukoplakia. This finding underscores the importance of thorough clinical examination and histopathological analysis in patients with risk factors for oral lesions, as emphasised by Gill *et al* [15].

Certainly, the patient’s history of tobacco use merits special consideration in the context of this atypical OVX presentation. While OVX is not directly associated with tobacco use, the habit may have contributed to the lesion’s unusual appearance as white plaques, initially mimicking leukoplakia. This underscores the complex interplay between tobacco use and oral mucosal changes, as noted by Reibel [16] in his comprehensive review of tobacco and oral diseases. The presence of tobacco-induced alterations could potentially mask or modify the typical clinical features of OVX, leading to diagnostic challenges. This case highlights the importance of considering a broad differential diagnosis, including both tobacco-related and non-tobacco-related lesions, in patients with a history of tobacco use presenting with atypical oral mucosal changes.

The management of our case through conservative excision aligns with the standard treatment approach for OVX. This is supported by literature reporting low recurrence rates, ranging from 3% Belknap *et al* [3] to 12% Tamiolakis *et al* [2] in large case studies.

Interestingly, our patient’s lipid profile showed slightly low VLDL levels, a finding not commonly reported in OVX cases. This observation may warrant further investigation into the potential relationship between lipid metabolism and OVX development.

In conclusion, our case contributes to the growing body of literature on OVX by highlighting its potential to mimic other white lesions, particularly when occurring in atypical locations. It underscores the critical role of histopathological examination in accurate diagnosis and emphasises the need for clinicians to maintain a high index of suspicion for OVX, even when presenting with unusual clinical features.

## Conclusion

The presentation of OVX on the labial mucosa as leukoplakia in a patient with a history of tobacco use underscores the need for careful evaluation, accurate diagnosis and appropriate management. While VX itself is benign, it is essential to rule out any co-existing conditions, especially in atypical presentations.

In conclusion, the collective insights from these studies and case reports have enhanced our understanding of OVX, its clinical presentations, potential associations and pathogenesis. OVX remains a rare but diagnostically significant oral lesion. This discussion highlights the importance of continued research in recognising, diagnosing and managing OVX effectively. It also emphasizes the need for a multidisciplinary approach involving clinicians, pathologists and surgeons to provide the best care to affected patients.

## Conflicts of interest

The authors declare that they have no conflicts of interest.

## Funding

No funding was received for the current case report.

## Figures and Tables

**Table 1.** Review of case series.

.	Year	Author	Age	Sex	Morphology	No. of cases	Site
1	1971 [1]	Shafer	Range from 27 to 67 years	Eight in women and seven in men.		15	Lower alveolar ridge, upper surface of a mandibular torus., lingual aspect of the mandible, palate and the floor of the mouth, lip and lower mucobuccal fold.
2	1996 [17]	Jehn-Shyun Huang, Chuen-Chyi Tseng, Ying-Tai Jin, Chi-Chou Huang, Tong-Yiu V. Wong, Hung-An Chen, Hong-Rong Chen, Mark Yen-Ping Kuo, Ying-Shiung Kuo	67,35	Male	Papillary	2	Lingual mucosa
3	2003 [18]	Philipsen HP, Reichart PA, Takata T, Ogawa I.	Females (mean age, 54.9 years) and males (mean age, 44.2 years)		Sessile or pedunculated base is a red/pink, papillary/granular/verrucous mucosal growth	282	Gingival margin and other areas of the masticatory oral mucosa
4	2005 [11]	Hu JA, Li Y, Li S.	43,48,69	Male	Plaque, hyperplastic, verruciform	3	Buccal mucosa,gingiva, palate.
5	2007 [4]	Yu CH, Tsai TC, Wang JT, Liu BY, Wang YP, Sun A, et al	45 years (range, 18–79 years).	Eight male and seven female patients.	Verrucous type, three (20%) the papillary type, and five (33%) the flat type.	15	Seven cases occurred on the gingiva, four on the tongue, and four on the buccal or vestibular mucosa
6	2017 [5]	Santiago E, Rosebush MS, Owens J, Cordell KG	Mean age was 56.6	Males made up 57.3%	-	90	Most common location was the gingiva (47.8%) with 58.1% of the gingival lesions occurring in the mandible
7	2018 [2]	Paris Tamiolakis, Vasileios,Theofilou, Konstantinos, Tosios, Alexandra Sklavounou-Andrikopoulou published	Mean age of 48.8 ± 14 years	8 males and 5 females	Mostly on gingiva hard palate.	13	Nodules or plaques
8	2019 [6]	Barrett AW, Boyapati RP, Bisase BS, Norris PM, Shelley MJ, Collyer J, *et al*	Mean age = 54.5 years	6 men and 5 women	Florid, polypoid, and carcinomatous	11	Tongue, commissure
9	2020 [3]	Belknap AN, Islam MN, Bhattacharyya I, Cohen DM, Fitzpatrick SG	Mean age of 61 years (range of 9–94),	Female: male ratio of 1.06:1.	Most frequently pink in colour, and most often described as bumpy, rough, verrucoid and/or papillary	212	Gingiva (*n* = 110), followed by palate (*n* = 41), buccal mucosa (*n* = 18), tongue (*n* = 20, ), vestibule (*n* = 13 lip (*n* = 4), floor of mouth (*n* = 3), and unspecified *n* = 1
10	2022 [19]	Aihie OP, Azzam MJ, Haroon A, Braudis K.	Two cases described in detail which were covid positive	Polypoid	18 (orofacial lesions)	Cutaneous involvement of lip and other tissues.	
